# LIDAL, a Time-of-Flight Radiation Detector for the International Space Station: Description and Ground Calibration

**DOI:** 10.3390/s23073559

**Published:** 2023-03-28

**Authors:** Giulia Romoli, Luca Di Fino, Giorgia Santi Amantini, Virginia Boretti, Luca Lunati, Carolina Berucci, Roberto Messi, Alessandro Rizzo, Pietro Albicocco, Cinzia De Donato, Giuseppe Masciantonio, Maria Cristina Morone, Giovanni Nobili, Giorgio Baiocco, Alice Mentana, Marco Pullia, Francesco Tommasino, Elisa Carrubba, Antonio Bardi, Marco Passerai, Dario Castagnolo, Gabriele Mascetti, Marino Crisconio, Livio Narici

**Affiliations:** 1 Department of Physics, University of Rome Tor Vergata, 00133 Rome, Italy; 2 National Institute for Nuclear Physics (INFN), University of Rome Tor Vergata, 00133 Rome, Italy; 3 Italian Space Agency (ASI), 00133 Rome, Italy; 4 Institute of Radioprotection (IRP), Italian National Agency for New Technologies, Energy and Sustainable Economic Development (ENEA), 00123 Roma, Italy; 5 National Institute for Nuclear Physics—Frascati National Laboratory, 00044 Rome, Italy; 6 Department of Physics, University of Pavia, 27100 Pavia, Italy; 7 The National Center for Oncological Hadron-Therapy (CNAO), 27100 Pavia, Italy; 8 Department of Physics, University of Trento, 38123 Trento, Italy; 9 Kayser Italy, 57128 Livorno, Italy; 10 Telespazio, 80125 Napoli, Italy

**Keywords:** space radiation, detector calibration, time-of-flight measurement

## Abstract

LIDAL (Light Ion Detector for ALTEA, Anomalous Long-Term Effects on Astronauts) is a radiation detector designed to measure the flux, the energy spectra and, for the first time, the time-of-flight of ions in a space habitat. It features a combination of striped silicon sensors for the measurement of deposited energy (using the ALTEA device, which operated from 2006 to 2012 in the International Space Station) and fast scintillators for the time-of-flight measurement. LIDAL was tested and calibrated using the proton beam line at TIFPA (Trento Institute for Fundamental Physics Application) and the carbon beam line at CNAO (National Center for Oncology Hadron-therapy) in 2019. The performance of the time-of-flight system featured a time resolution (sigma) less than 100 ps. Here, we describe the detector and the results of these tests, providing ground calibration curves along with the methodology established for processing the detector’s data. LIDAL was uploaded in the International Space Station in November 2019 and it has been operative in the Columbus module since January 2020.

## 1. Introduction

One of the major risks for the health and performance of astronauts during upcoming deep space flights (return to the Moon, Mars and beyond) is exposure to space radiation [1,2]. Away from Earth’s magnetic field, astronauts will be mostly vulnerable to galactic cosmic rays (GCRs) and to the radiation associated with transient solar events (SPEs, solar particle events). Within this context, the characterization of the radiation environment in the spacecraft is needed to estimate risks and develop proper countermeasures. Furthermore, it supports the validation of radiation and transport models.

Area radiation detectors can provide detailed radiation measurements to support advanced risk models [3]. The physical and radiation protection parameters introduced so far have proven to be of increasing complexity. They went from the dose, which is measurable even with a single small slab of silicon, to the equivalent dose, for which the measurement of the energy deposited for unit of pathlength (LET, linear energy Transfer) is needed, and therefore requires a device capable of tracking. Recent risk assessment models require more information about incoming radiation. The NASA model, for example, is based on the ratio Z^2^/β^2^ (Z: ion charge and β = v/c, v: velocity of the particle, c: speed of light in vacuum) [4]. It is conceivable that separate information of Z and β (nuclear identification) will further improve risk assessment capabilities.

LIDAL provides the needed information for the evaluation of both Z and β in a volume still compatible for use in a spacecraft/base such as the International Space Station (ISS). This is accomplished by measuring the time-of-flight (ToF) of the detected ions (featured for the first time in a space habitat). In this paper, we present the results from the LIDAL calibration campaign at two particle accelerator facilities in 2019. Furthermore, a detailed description of the detector, its mechanics and electronics is provided.

## 2. Materials and Methods

The LIDAL detector (Light Ion Detector for ALTEA-Anomalous Long-Term Effects on Astronauts) [5,6,7,8,9] is an active particle detector of compact dimensions (19 × 25 × 54 cm^3^) now operating in the International Space Station (ISS). It is an upgrade of the ALTEA detector system [10,11,12], which measured the radiation on-board the ISS in the 2006–2012 period. The ALTEA system returned to Earth in 2014 and was successfully tested at the TIFPA accelerator facility to check its proper functioning in 2016. LIDAL has been manifested as a re-flight of ALTEA, so the same interface with the station had to be maintained. To accomplish this, one of the challenges in designing LIDAL was to link the new electronics with the ALTEA one.

**Figure 1:**
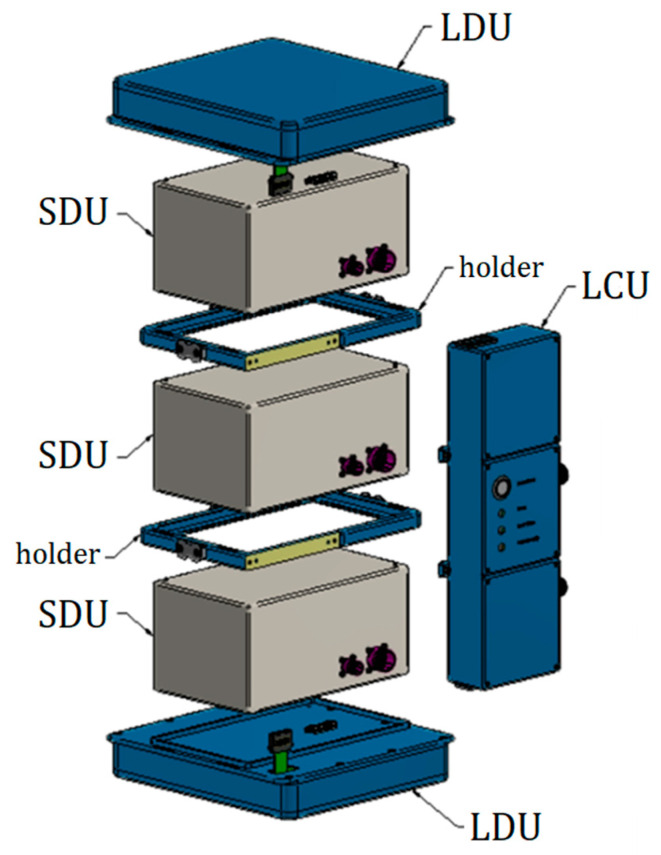
Sketch of the LIDAL detector. It consists of two LIDAL Detector Units (LDUs), containing fast timing scintillator bars and placed 49 cm apart, and three Silicon Detector Units (SDUs) from ALTEA. The LIDAL Collector Unit (LCU) and the data acquisition unit (DAU) (here not shown) manage data acquisition.

LIDAL enhances the particle detection capabilities of its precursor. This is accomplished by stacking three of ALTEA Silicon Detector Units (SDUs) in a tower configuration and placing them between two arrays of plastic scintillators (hereafter LDUs, LIDAL Detector Units) (Figure 1). Each SDU is a six-plane striped silicon telescope able to track and measure energy releases of all passing through ions (Z > 2, and protons and helium ions in the ~25–45 MeV and ~25–250 MeV/n energy windows respectively). The 3-SDU tower extends the ion discrimination capability of ALTEA, featuring 18 silicon planes. Furthermore, the LDU subsystems perform time-of-flight measurements, and these add information about the incident particle, supplementary to the energies deposited in the silicon planes and measured by the SDUs. In this way, ion discrimination is enhanced, and by comparing released energies and ToF measurements with results from simulations, it is possible to evaluate the charge of the traversing particle and its kinetic energy before entering the detector. The calibration of both SDUs and ToF systems is therefore a needed and sufficient step to validate this estimation. Finally, the integration between SDUs and scintillator planes provides trigger signals to expand the acceptance window for light nuclei, such as protons and helium ions, resulting in LIDAL being sensitive to the whole ion spectra.

In sum, the LIDAL detector is sensitive to the full ion spectra, thanks to the scintillators. The lower sensitivity of the SDUs is limited to a LET of 2.3 keV/µm, as described below.

### 2.1. SDU

A single SDU is a six-plane particle telescope. Each plane consists of 8 × 8 cm^2^ silicon wafers, 380 µm thick, placed side by side on an aluminum support and spaced 5.5 mm apart. Each silicon wafer is segmented into 32 strips, with a pitch of 2.5 mm. Strip segmentation is alternatively oriented in the two orthogonal directions (X and Y) between successive planes, in order to use the position of the hit strips for particle track reconstruction. The distance between an X plane and the correspondent Y plane is equal to 3.75 mm, while the distance between each XY pair is 37.5 mm. The outer box of each SDU is made of 1.3 mm thick aluminum and the whole structure results in a double-ended geometrical factor of 230 cm^2^ sr (corresponding to a field of view that ranges between 0°and 62°) [13,14].

The silicon chips in the SDU were deployed in the SilEye detectors [15,16] and originally used for the NINA (New Instrument for Nuclear Analysis) cosmic ray space telescopes [17]. The charge released in the silicon strips by incoming particles is processed by an amplification chain consisting of a charge-sensitive amplifier (CSA), a forming amplifier, a track-and-hold circuit and a switch. The output analogical signals coming from the amplification stage are then sent to a multiplexer that selects and sequentially sends the data to a 12-bit analog to digital converter (ADC), placed in the ROE (Read Out Electronics) board. The energy resolution is 0.64 MIP (minimum ionizing particle, 1 MIP = 109 keV in 380 μm silicon)/ADC channel and the energy loss of the particles in each plane ranges from 10 to 2400 MIP (molybdenum is the heaviest detectable nuclear species, as it releases energy close to saturation (2000 MIP) at minimum energy loss (at 2 GeV/n)) [13], with 10 MIP (about 3 keV/µm) as the threshold value for triggering the silicon plane. A data buffer is built by sending ROE data to a FIFO (first-in first-out) register. Data are then gathered by the data acquisition unit (DAU), which collects, organizes and distributes them in packets, adding time labels.

### 2.2. LDU and LCU

The two LDUs of the LIDAL detector measure the particle ToF with a targeted resolution better than 120 ps and are positioned 49 cm apart. In this way, the LIDAL detector results in a double-ended geometrical factor of 13.5 cm^2^ sr (corresponding to a field of view that ranges between 0° and 18°).

**Figure 2:**
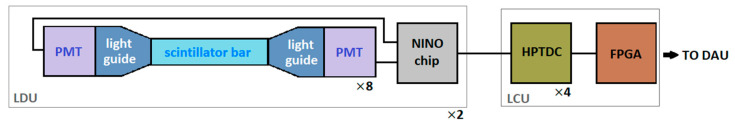
Sketch of the LIDAL components devoted to the processing of signals coming from the scintillator bars. Each LDU is composed of eight plastic scintillators, optically coupled with photomultipliers. Signals from 16 PMTs (one LDU) are processed by 2 NINO chips. The LCU manages data coming from both LDUs and interfaces with the DAU. 4 HPTDCs convert signals from NINO chips into time signals, which are then sent to an FPGA, then processed and sent out to the DAU.

Figure 2 summarizes the main components that process signals coming from the scintillator bars, leading to time measurements. Each LDU is an independent detection plane, made up of eight EJ-230 plastic scintillators for fast timing application [18], optically coupled with photomultipliers (PMT) through plastic light guides. The size of each scintillator (80 × 20 × 4 mm^3^) was chosen to obtain a compromise between the need for a large signal to generate the trigger and a quick temporal response. At each of the two ends of a plastic scintillator, a light guide conveys the scintillation light toward the input window of a PMT Hamamatsu RS9880-U110 [19], which transforms the light pulse into an electric current signal. Scintillator bars and light guides are wrapped in aluminized mylar foil to maximize the light collected at the extremities. The scintillators are wrapped in black tape to ensure that the system is light-tight. Signals from 16 PMTs (on one LDU) are processed by 2 NINO (fast pre-amplifiers and discriminator devices developed by CERN) chips (one per side) [20]. The signals coming from the NINO (New Integrated circuit for the NOn-imaging cherenkov detector) chips are then sent to 4 HPTDC (high performance TDC (time to digital converter)) chips [21], housed in the LIDAL Control Unit (LCU), which manages data coming from both LDUs and interfaces with the DAU. The HPTDC chips convert the differential inputs into time signals, with a time accuracy of 25 ps. The converted data are, finally, read by an ARTIX XC7a200tfgg484 FPGA (field-programmable gate array), processed and sent out to the DAU.

### 2.3. DAU

The DAU provides power to the detector and manages data synchronization, collection and transmission. When a trigger signal is produced from either a temporal coincidence of the scintillator planes (signals read from both LDUs are within the same time acceptance window) or an above-threshold signal from one SDU (the sum of the energy releases in a 4 strip group is greater than 3 keV/µm in all the X planes of one SDU), data from the different detector units are collected by the DAU, referred to as part of the same particle event, organized into multiple data packets (24-bit length), time-labelled and sent out.

### 2.4. Pre-Processing

Particle data from the three SDUs and the two LDUs are handled separately. Specifically, the pre-processing algorithm extracts information from the binary data and builds a coherent picture of each particle that has interacted with the LIDAL detector. This is accomplished through several analysis steps:

- Unpacking: Data from the binary stream are converted into a structured format, storing all times (coming from the scintillator bars) and released energies (coming from the silicon strips) for each particle event. LDU data require an additional step, namely the conversion of DAU data packets (24-bit length) into HPTDC data format (32-bit length).
- Calibration/reconstruction: Data from the silicon detectors must be calibrated and corrected (see next paragraph), and data from the scintillator bars must be combined to rebuild ToF (see Appendix A).
- Event selection: Recombined data must be checked to exclude incomplete (the accelerator rate was nominally kept below 102 Hz. However, the oscillations of the accelerator with these settings resulted in rates that could reach several hundred Hz. This high rate, focused on the same scintillators and silicon strips, could be a reason for incomplete events) and spurious events.

### 2.5. SDU Data Handling

When a particle crosses LIDAL, it releases energy in one or more silicon strips in the SDUs. The first step of the pre-processing analysis is the removal of the signal offset with null input (referred to as the pedestal) given by the temperature-dependent dark currents in the silicon strips. Pedestals are periodically acquired during LIDAL automatic calibration runs, in order to assess the detector baseline. A periodic calibration, consisting in 100 pedestals for each strip, is acquired by the DAU every hour or every 1-degree temperature increase. The average value of these pedestals is then subtracted from all the particle signals during data pre-processing. As already stated, for each strip hit by a particle, the system acquires the signal of at least 4 strips, and signals from the non-hit strips should be equal to zero. As already observed with the previous ALTEA system, their average value is generally negative. This value is therefore subtracted as a further correction to the standard pedestal.

The second step of the analysis is trajectory reconstruction. Given all the silicon energy releases detected for a particle event, calibrated and corrected, the maximum energy release for each single silicon plane is considered. The trajectory of the incident particle is then reconstructed by obtaining the space coordinates of the strips where maxima energy releases were detected. The alignment of the track is thus evaluated, and the algorithm calculates the coefficients of the straight line that represents the trajectory of the incident particle according to the method of least squares.

### 2.6. LDU Data Handling

The time-of-flight of a particle is computed from the combined information of the two LDUs. The ToF of particles traversing the whole LIDAL detector and activating both LDUs was evaluated according to the following formula:

(1) $$\mathrm{ToF}=\frac{t_{1}+t_{2}+d_{12}}{2}-\frac{t_{3}+t_{4}+d_{34}}{2}$$

where (*t*_1_, *t*_2_) and (*t*_3_, *t*_4_) are the minimum times recorded on both ends of the scintillators hit by the incident particle (upstream and downstream LDUs, respectively), and *d*_12_ and *d*_34_ are two constant terms which account for the different delays with which temporal signals on the sides of the same scintillator are read out. As a very first approximation, *d*_12_ = *d*_34_. On the other hand, from the flight measurements it has been measured that the differences between these constant terms cannot be neglected in LIDAL, leading to a correction of the ToF value of at most 12%. This correction is applied in the following, and more details are provided in Appendix A and Appendix B.

During the analysis of LIDAL data, we found spurious events (referred to as “ghosts” (following the nomenclature used in [22] when describing the performance of the Fermi Large Area Telescope in orbit). Ghost events from LIDAL are correlated with the overlap of time acquisition windows, so that they can be identified and selected out. The PMTs’ signals of a LIDAL event correspond to the passage of a particle j. Indeed, the detector opens an acceptance window at the passage of the particle. All temporal signals within this window are associated with the event and read with respect to the time origin of the window, equal to the time T(j) at which the acceptance window was opened. In principle, acceptance windows associated with different sampling signals are not expected to overlap with each other. On the other hand, it has been observed that LIDAL acceptance windows do overlap, and the same temporal signals may be read several times, with respect to different time origins. Each LIDAL event containing times associated to the previous time window were excluded from the analysis.

## 3. Results

**Figure 3:**
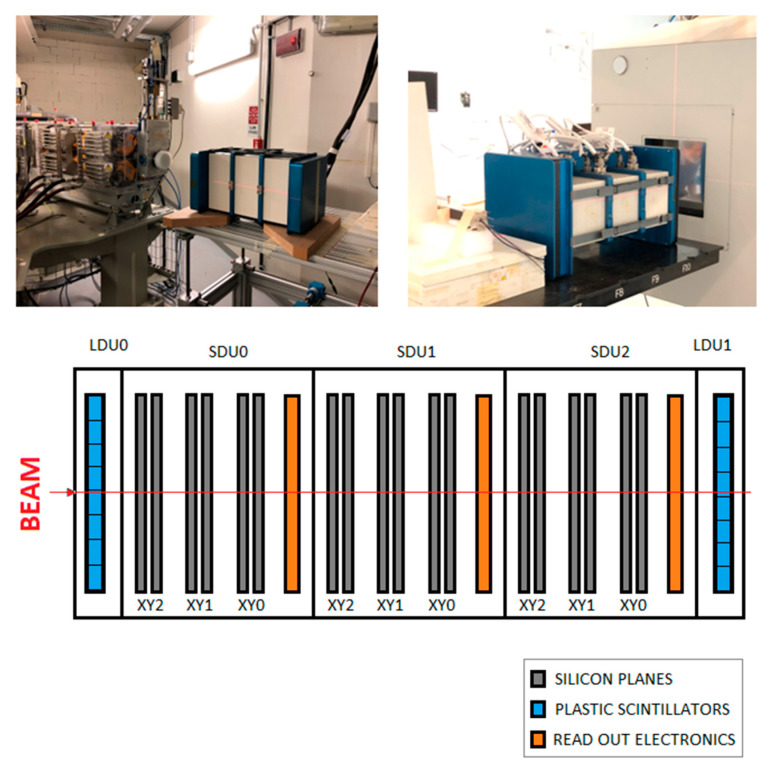
Session measurements at TIPFA (**left**) and CNAO (**right**) particle accelerator facilities and a sketch of the experimental set up (dimensions of components are not in scale); the names of the components of LIDAL (SDUs and LDUs) are specified. On the bottom of the figure, the names of the planes are specified (note that XY2 refers to the couple of planes X2 and Y2, the same for the others).

Tests of the LIDAL detector were performed over several measurement sessions at the TIPFA (Trento Institute for Fundamental Physics Application; Trento, Italy) and CNAO (National Center for Oncology Hadron-therapy; Pavia, Italy) particle accelerator facilities in June 2019. At TIPFA, LIDAL was irradiated with protons at 90, 110, 130, 170 and 220 MeV. At CNAO, the detector was tested with carbon ions at 180, 200, 300 and 400 MeV/n. Figure 3 shows the experimental setup. All measurements were performed with the beam perpendicular to the sensitive planes of the detector, and the beam particles first crossed the silicon planes and then the ROE of each SDU. Moreover, the position of the detector with respect to the beam was shifted throughout the various measurements along the longitudinal direction to check the response of all the plastic scintillators.

### 3.1. The Time-of-Flight Measurements (from LDUs)

**Figure 4:**
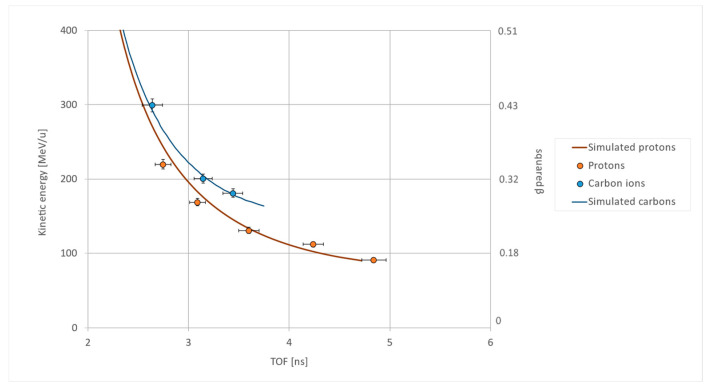
ToF measurements (in ns) made at TIPFA (proton beam line, orange dots) and CNAO (carbon beam line, blue dots). The solid lines result from the simulation of protons (orange line) and carbon ions (blue line) traversing the detector; they are derived from a solution of the Bethe-Bloch equation (see text). The consistency between measured values and simulation results confirms LIDAL ToF measurements and its consequent capability in evaluating β. For the measurement session with carbon ions at 400 MeV/n, only SDU data were acquired.

**Figure 5:**
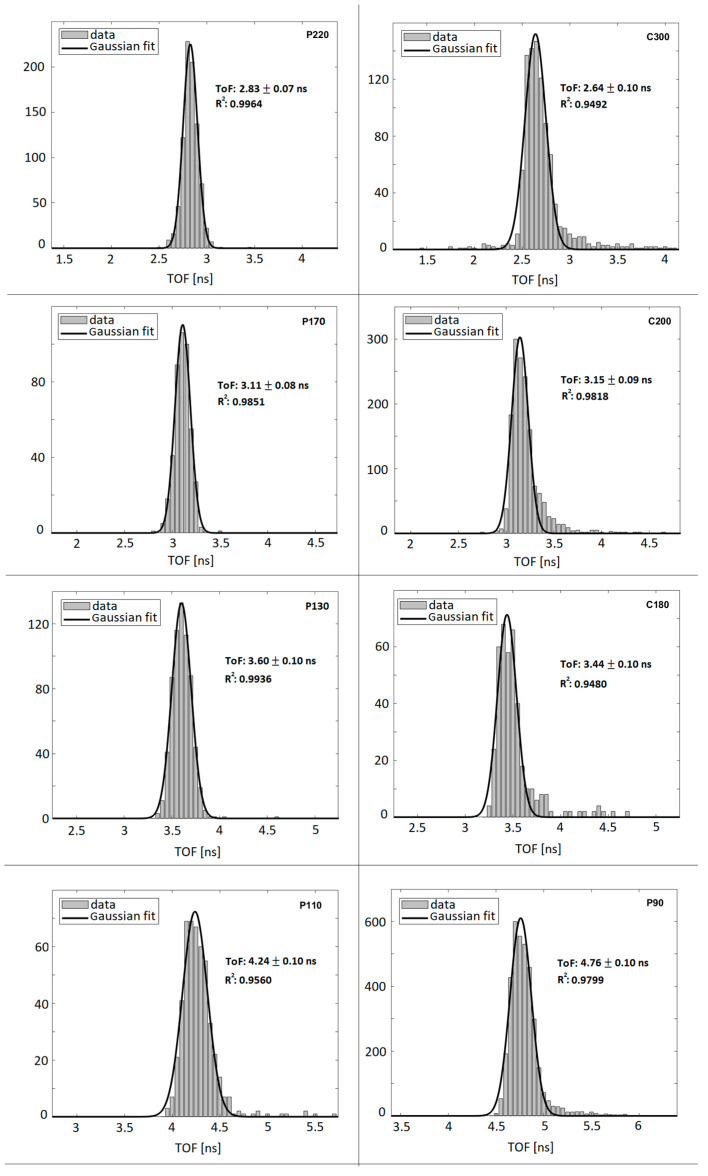
ToF distributions (in ns) with Gaussian fits superimposed for protons and carbon ions at different energies. Time resolution has proven to be between 70 and 100 ps, regardless of the scintillators hit by the particle beam.

Figure 4 and Figure 5 show the results for the ToF measurements. The time-of-flight of protons and carbon ions at different energies was computed using Equation (1), where the times t_i_ are the values read on both sides of the two scintillator bars hit by the particle beam. The widths of the Gaussian functions used to fit the time measurements ranged between 70 and 100 ps for all the scintillators, confirming an overall temporal resolution of the detector lower than the targeted value of 120 ps. The time-of-flight measurement is relative to particles traversing the many layers of the detector. The energies released inside LIDAL have to be taken into account in the estimation of the particle’s input kinetic energy. To this end, a solution of the Bethe-Bloch equation as derived in [23,24] was exploited to simulate the time a particle of known charge and input kinetic energy needs to traverse the LIDAL detector. Nominal kinetic energies and the results from this simulation are always compatible.

### 3.2. The Delivered Energy Measurements (from SDUs)

**Figure 6:**
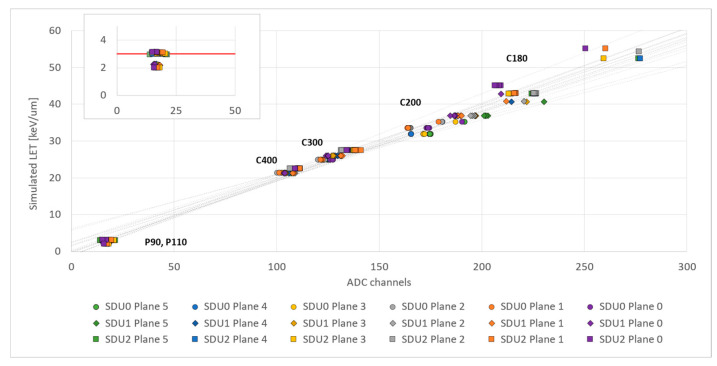
Simulated particles’ LET/plane (in keV/µm) vs. experimental data (in ADC) for all the silicon planes of the three SDUs. Linear fits are displayed to demonstrate the variability between different planes. A low-LET blow-up is shown to demonstrate the sensitivity of the detector below the threshold value of 3 keV/µm (red dashed line). Note that measurements for C200 and C180 in the different silicon planes partially overlap.

**Figure 7:**
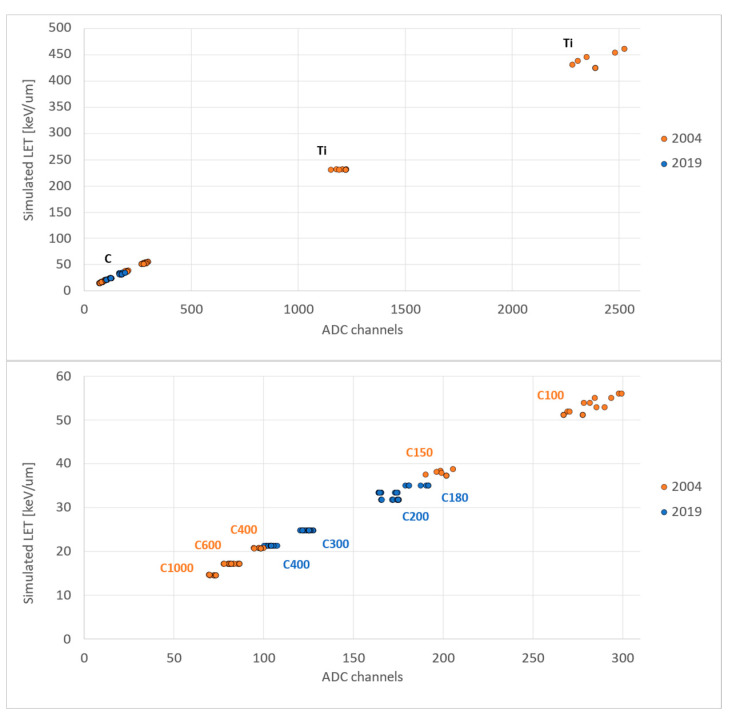
Simulated particles’ LET/plane vs. experimental data for all the silicon planes of SDU0 from the 2019 measurement session at CNAO (blue dots) and 2004 measurement sessions at GSI-Darmstadt (orange dots).

Regarding the performance of the SDUs, average energy releases per plane were measured for each session with protons and carbon ions. After pedestal subtraction and correction of negative values, the mean energy release from each silicon plane was analyzed and the peak position estimated by a Gaussian fit. The experimental data were then compared with results from simulations to obtain calibration curves (Figure 6). Monte Carlo simulations (Monte Carlo radiation transport code PHITS (version 3.22) [25]) reproducing the experimental setup, beam setting and all interactions were performed to simulate the behavior of particles of known charge and input energy (protons at 220, 170, 110 and 90 MeV, and carbon ions at 180, 200, 300 and 400 MeV/n) inside the detector. The simulation provides the statistical distributions for the needed physical parameters. In particular, for each beam energy of protons or carbon ions, simulations provided the dose distribution of the linear energy transfer (LET), whose first moment is the dose average LET (indicated with Simulated LET in Figure 6 and Figure 7). The comparison between measured and simulated average energy releases per plane results in the conversion factors (when SDU0 was the first SDU hit by the particle beam, proton energy releases in the SDU0 silicon planes could not be measured, as they were below the sensitivity of the detector. On the other hand, proton energy releases in the SDU1 and SDU2 silicon planes could be measured, even if below or very close to the SDU threshold of 3 keV/µm, as discussed below) listed in Table 1.

#### Comparison with Previous ALTEA Calibration

The results from these measurement sessions were compared with those obtained from the two ALTEA flight model beam tests performed at GSI-Darmstadt in November 2003 and April 2004 [13,14] as shown in Figure 6. In the November 2003 beam test, the calibration of SDU0-FM was performed using carbon nuclei at different energies: 100, 150, 400 and 600 MeV/n (bottom panel). In the April 2004 beam test, the full ALTEA FM was calibrated with carbon nuclei (beams at 100, 600 and 1000 MeV/n) and titanium nuclei (beams at 200 and 600 MeV/n) (top panel). The experimental results at that time were compared with GEANT3 Monte Carlo simulations of individual SDUs, to obtain the conversion factors listed in Table 2.

## 4. Discussion

In this work, the LIDAL particle detector has been presented, together with the results from the tests performed at two particle accelerator facilities in 2019.

### 4.1. Time-of-Flight Measurements

ToF measurements show a time resolution (from 70 to 100 ps) lower than the 120 ps threshold that was targeted during the detector’s development. The ToF distributions are mostly Gaussian, however, especially for carbon ions, they show non-Gaussian tails. These could be explained by the straggling of beam particles through the crossed materials.

### 4.2. SDU Calibration

Calibration curve results are linear for all planes (correlation factor R^2^ from 0.982 to 0.999) and provide the keV/µm vs. ADC channels conversion factors summarized in Table 2. The integration between SDUs and scintillator planes enhances LIDAL sensitivity to protons, as expected (Figure 5, inset). Measured energy releases of protons in the last silicon planes hit by the beam particles can be noticed below the 3 keV/µm threshold needed to start an SDU auto-trigger. The trigger offered by the scintillators makes LIDAL more sensitive than its predecessor for detecting protons, being able to discriminate particles with single plane energy releases down to 2.3 keV/µm.

### 4.3. Comparison with Previous Calibrations

The results for the SDUs show a remarkable agreement with the calibrations performed about 15 years ago (Figure 6), despite the long time spent by the telescopes on board the ISS in the 2006–2012 period, and the download-upload of the detector from and to the ISS.

Finally, flight measurements [9] were used as a further support of ground calibrations to define some of the parameters needed in data pre-processing (see Appendix A and Appendix B). This is an example of how the detector’s calibration and pre-processing can be further improved, even during flight operations.

LIDAL is currently operating in the Columbus module onboard the ISS and is scheduled to monitor the radiation space environment at least until 2024.

## Figures and Tables

**Table 1:** Conversion factors (keV/µm vs. ADC channels) for the SDU0, SDU1 and SDU2 silicon planes. Differences among different planes are within 10%.

SDU	Plane Y0	Plane X0	Plane Y1	Plane X1	Plane Y2	Plane X2
SDU0	0.18 ± 0.01	0.19 ± 0.01	0.18 ± 0.01	0.15 ± 0.01	0.18 ± 0.01	0.15 ± 0.01
SDU1	0.20 ± 0.01	0.20 ± 0.01	0.19 ± 0.01	0.18 ± 0.01	0.20 ± 0.01	0.18 ± 0.01
SDU2	0.22 ± 0.01	0.21 ± 0.01	0.19 ± 0.01	0.21 ± 0.01	0.19 ± 0.01	0.19 ± 0.02

**Table 2:** The same as Table 1, but from the calibration campaigns performed at GSI-Darmstadt in November 2003 and April 2004.

SDU	Plane Y0	Plane X0	Plane Y1	Plane X1	Plane Y2	Plane X2
SDU0	0.18 ± 0.01	0.18 ± 0.01	0.19 ± 0.01	0.19 ± 0.01	0.19 ± 0.01	0.18 ± 0.01
SDU1	0.18 ± 0.01	0.19 ± 0.01	0.19 ± 0.01	0.18 ± 0.01	0.18 ± 0.01	0.19 ± 0.01
SDU2	0.17 ± 0.01	0.18 ± 0.01	0.17 ± 0.01	0.19 ± 0.01	0.17 ± 0.01	0.18 ± 0.01

**Figure A1:**
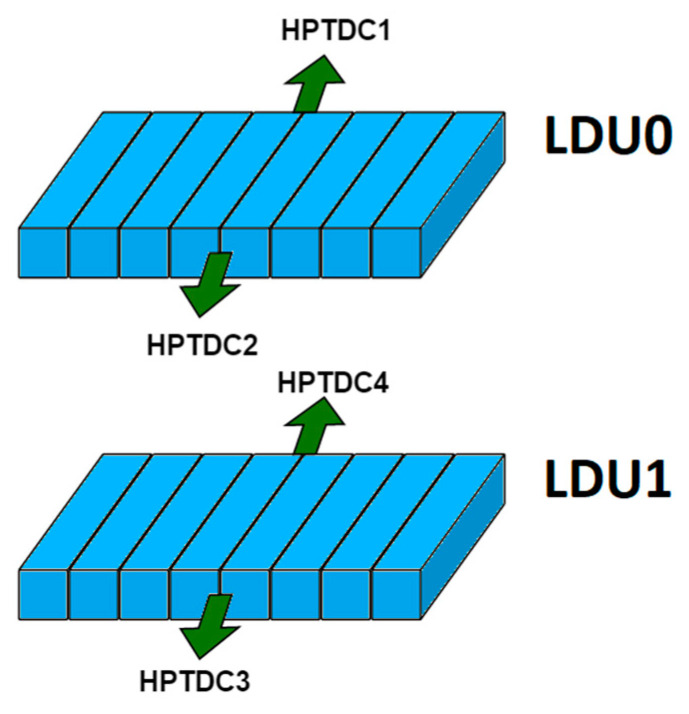
Numbering of the HPTDCs that read out signals from the scintillator bars of the LIDAL detector. Each HPTDC processes signals coming from the same ends of the scintillators of an LDU.

**Figure A2:**
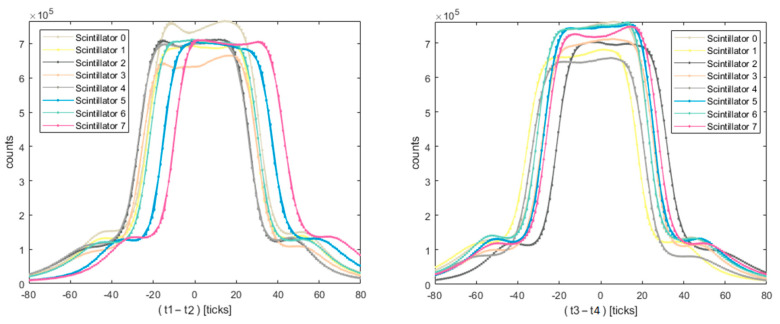
Distributions of time differences read out from the ends of LIDAL plastic scintillators (**left** and **right** for LDU0 and LDU1, respectively), derived from about two years of flight data. Distributions are not zero-centered, and temporal corrective terms must be introduced.

## Data Availability

Not applicable.

## Appendix A

This section will explain the handling of temporal signals coming from LIDAL LDUs.

Take a particle that traverses both LIDAL scintillating planes. At the sampling signal, a trigger matching window is opened and all PMTs’ temporal signals from the trigger time inside the window are read out. Each particle *P* is then associated with a set of times:

(A1) $$P=\left\{t_{i,k}\right\},$$

where *i* = 0, …, 31 refers to the *i*th PMT of LIDAL and *k* = 1, …, *m_i_* to the multiple times read from it, with *m_i_* as its multiplicity (in the ideal case, each PMT outputs a single temporal signal, *m_i_* = 1, and 4 PMT signals per particle event are read out. On the contrary, it has been observed that more than 4 PMTs often output multiple temporal signals when a particle traverses the detector). For each particle, the set of times *P* is divided into four subsets by considering the times read from the same side of the scintillators of an LDU, that is, by considering the times given by the same HPTDC (Figure A1). Then, for each subset, the minimum time is selected. A particle event is congruous if the minimum times recorded on one LDU are on the same scintillator, and the same condition is fulfilled for both LDUs. If this is the case, the particle event is selected and particle ToF can be computed as:

(A2) $$\mathrm{ToF}=\frac{t_{1}+t_{2}}{2}-\frac{t_{3}+t_{4}}{2}$$

where *t_i_* is the minimum time recorded on the *i*th HPTDC. Equation (A2) does not take into account delays coming from light transmission, cable length and signal shape. In general, the two times *t*_1_ and *t*_2_, read at the ends of the same scintillator, are defined as:

(A3) $$t_{1}=t_{0}+\frac{x}{v_{eff}}+c_{1}$$

(A4) $$t_{2}=t_{0}+\frac{L-x}{v_{eff}}+c_{2}$$

where *L* is the scintillator length of 8 cm, *v_eff_* is the effective velocity of photons inside the scintillating material (about 13 cm/ns), and *c*_1_ and *c*_2_ are two constants which take into account the time needed by the signal to travel in the PMT and through the threads of the HPTDC (in general, *c*_1_ ≠ *c*_2_). It has been observed that such corrective terms cannot be neglected for the LIDAL detector, and the particle ToF has to be estimated as:

(A5) $$\mathrm{ToF}=\frac{t_{1}+t_{2}+d_{12}}{2}-\frac{t_{3}+t_{4}+d_{34}}{2}$$

where *d*_12_ = (*c*_1_ − *c*_2_) and *d*_34_ = (*c*_3_ − *c*_4_), two constant terms specific for the two scintillators hit by the particle, which account for the different delays with which temporal signals on the sides of the same scintillator are read out. All corrective terms were estimated from about two years of LIDAL flight data, as explained below, and applied to the temporal signals measured on the ground, so to calculate ToF as in Equation (A5). Note that Equation (A5) is the same as Equation (1).

## Appendix B

This section will explain how the corrective terms introduced in Appendix A were calculated. Take a particle that traverses one LIDAL plastic scintillator. Given the two times *t*_1_ and *t*_2_ measured at the two scintillator ends, the difference between them gives an estimate of the point of impact x of the particle on the scintillator:

(A6) $$x=\frac{v_{eff}}{2}\left(t_{1}-t_{2}\right)+\frac{v_{eff}}{2}\left(L+c_{2}-c_{1}\right)=\frac{v_{eff}}{2}\left(t_{1}-t_{2}\right)+C$$

Suppose that n particles (*n → ∞*) are hitting the same scintillator. In the case where the times at the scintillator ends are read out without temporal delay, one expects that the distribution of all times’ differences (*t*_1_ − *t*_2_) is centered on zero, and that the maximum time difference corresponds to the time a photon needs to traverse the whole scintillator length. This distribution was derived from flight data (for these corrections, the data acquired in flight were used. Indeed, it was necessary to acquire high particle event statistics over the full length of each scintillator. This requirement was not fulfilled by ground measurements) for each LIDAL plastic scintillator, and it was realized that it was not centered on zero for most of them (Figure A2). This can be justified by the fact that there are different delays in reading times at the ends of the same scintillator. Therefore, corrective terms were introduced, defined as follows:

(A7) $$\begin{array}{c} t_{1}^{new}=t_{1}\\ t_{2}^{new}=t_{2}+\left(c_{1}-c_{2}\right)=t_{2}+d_{12} \end{array}$$

where *d*_12_ = (*c*_1_ − *c*_2_) is a constant term specific for a single scintillator that takes into account the fact that times read from one scintillator’s side are delayed with respect to the times read out from the other side. All constant terms were derived by measuring the shift from zero of the centers of the scintillators’ distributions of right/left time differences and applied to LIDAL measured temporal signals. These constant terms are also used to track the particle. From the position of the hit channel and from the difference of the times read at the ends of the scintillator bar, one can reconstruct the hit position on the top and bottom scintillator layers.
